# Regorafenib combined with PD1 blockade increases CD8 T-cell infiltration by inducing CXCL10 expression in hepatocellular carcinoma

**DOI:** 10.1136/jitc-2020-001435

**Published:** 2020-11-24

**Authors:** Kohei Shigeta, Aya Matsui, Hiroto Kikuchi, Sebastian Klein, Emilie Mamessier, Ivy X Chen, Shuichi Aoki, Shuji Kitahara, Koetsu Inoue, Ayako Shigeta, Tai Hato, Rakesh R Ramjiawan, Daniel Staiculescu, Dieter Zopf, Lukas Fiebig, Gabriela S Hobbs, Alexander Quaas, Simona Dima, Irinel Popescu, Peigen Huang, Lance L Munn, Mark Cobbold, Lipika Goyal, Andrew X Zhu, Rakesh K Jain, Dan G Duda

**Affiliations:** 1 Edwin L. Steele Laboratories for Tumor Biology, Department of Radiation Oncology, Massachusetts General Hospital, Boston, Massachusetts, USA; 2 Institute of Pathology, University Hospital Cologne, Cologne, Germany; 3 Drug Discovery, Bayer Pharma AG, Berlin, Germany; 4 Department of Medicine, Massachusetts General Hospital, Boston, Massachusetts, USA; 5 Center for General Surgery and Liver Transplantation, Clinical Institute Fundeni, Bucharest, Romania

**Keywords:** liver neoplasms, programmed cell death 1 receptor, drug therapy, combination

## Abstract

**Background and purpose:**

Combining inhibitors of vascular endothelial growth factor and the programmed cell death protein 1 (PD1) pathway has shown efficacy in multiple cancers, but the disease-specific and agent-specific mechanisms of benefit remain unclear. We examined the efficacy and defined the mechanisms of benefit when combining regorafenib (a multikinase antivascular endothelial growth factor receptor inhibitor) with PD1 blockade in murine hepatocellular carcinoma (HCC) models.

**Basic procedures:**

We used orthotopic models of HCC in mice with liver damage to test the effects of regorafenib—dosed orally at 5, 10 or 20 mg/kg daily—combined with anti-PD1 antibodies (10 mg/kg intraperitoneally thrice weekly). We evaluated the effects of therapy on tumor vasculature and immune microenvironment using immunofluorescence, flow cytometry, RNA-sequencing, ELISA and pharmacokinetic/pharmacodynamic studies in mice and in tissue and blood samples from patients with cancer.

**Main findings:**

Regorafenib/anti-PD1 combination therapy increased survival compared with regofarenib or anti-PD1 alone in a regorafenib dose-dependent manner. Combination therapy increased regorafenib uptake into the tumor tissues by normalizing the HCC vasculature and increasing CD8 T-cell infiltration and activation at an intermediate regorafenib dose. The efficacy of regorafenib/anti-PD1 therapy was compromised in mice lacking functional T cells (*Rag1*-deficient mice). Regorafenib treatment increased the transcription and protein expression of CXCL10—a ligand for CXCR3 expressed on tumor-infiltrating lymphocytes—in murine HCC and in blood of patients with HCC. Using *Cxcr3*-deficient mice, we demonstrate that CXCR3 mediated the increased intratumoral CD8 T-cell infiltration and the added survival benefit when regorafenib was combined with anti-PD1 therapy.

**Principal conclusions:**

Judicious regorafenib/anti-PD1 combination therapy can inhibit tumor growth and increase survival by normalizing tumor vasculature and increasing intratumoral CXCR3+CD8 T-cell infiltration through elevated CXCL10 expression in HCC cells.

## Background

Hepatocellular carcinoma (HCC) is a common cancer-related cause of death.^1 2^ HCC is an aggressive gastrointestinal cancer with increasing incidence in the USA, but existing therapeutic options have shown limited efficacy. HCCs are highly vascularized tumors, which often develop an arterialized blood supply that feeds tumor progression.^3^ Sorafenib—a broad tyrosine kinase inhibitor (TKI) with potent antivascular endothelial growth factor receptor (VEGFR)1–3 and platelet-derived growth factor receptor (PDGFR) activity—was the first systemic therapy to show increased overall survival (OS) in patients with advanced HCC in phase III trials.^4 5^ However, HCCs become resistant to sorafenib and the OS benefit is limited to approximately 3 months over placebo. Other broad TKIs—such as regorafenib and cabozantinib—and the anti-VEGFR2 antibody ramucirumab (in selected patients with high alpha-fetoprotein) also showed efficacy in phase III trials in patients with advanced HCC who progressed on sorafenib.^6–8^ Finally, the multitargeted TKI lenvatinib became a first-line therapy in advanced HCC after showing non-inferior survival compared with sorafenib.^9^ These successes argued for a key role of VEGF/VEGFR-driven angiogenesis in advanced HCC. However, other multitargeted TKIs—such as sunitinib, brivanib—or ramucirumab in unselected patients with HCC did not increase OS despite delaying tumor progression in phase III trials.^10^ This argues that the activity seen with the approved drugs may be in part mediated by inhibition of specific targets beyond VEGFRs and specific mechanisms of action.

HCCs usually develop in tissues affected by chronic inflammation and hypoxia due to underlying liver damage. This characteristic feature leads to immunosuppression via multiple mechanisms, including upregulation of the immune checkpoint molecules such as programmed cell death protein 1 (PD1) and its ligand programmed death-ligand 1 (PD-L1).^11–13^ Recently, immunotherapy strategies using immune checkpoint blockade (ICB) with anti-PD1 antibodies nivolumab and pembrolizumab alone as well as with nivolumab plus ipilimumab (an anticytotoxic T-lymphocyte antigen 4 antibody) received accelerated approval by the Food and Drug Administration; this was based on phase I/II trial data, which showed responses in 14%–31% of previously treated patients with advanced HCC, some of which were durable.^14 15^ However, PD1 blockade alone was insufficient to significantly increase OS in the randomized phase III trials conducted so far.

The limitations of monotherapy approaches in HCC led to the development of anti-VEGFR/PD1 combinations new therapies designed to address treatment resistance mechanisms and achieve synergy by increasing effector T-cell infiltration.^16^ One of the key elements of the efficacy of combinations of anti-VEGF with immunotherapies is maintaining a functional (normalized) vasculature and reducing hypoxia.^16–18^ We and others have demonstrated that blocking VEGF pathway can transiently normalize the tumor vasculature and increase cytotoxic T lymphocyte (CTL) infiltration, thus facilitating vaccine immunotherapy.^19^ Of note, recent studies showed that antitumor immune responses and vascular normalization can be reciprocally regulated by CD4+ T effector cells in breast cancers.^20^ In HCC, we recently demonstrated that dual VEGFR-2/PD1 blockade using antibodies normalizes tumor vasculature and induces antitumor immunity in mice with underlying liver damage.^21^

The clinical experience is consistent with preclinical findings. The use of a combination of the anti-VEGF antibody bevacizumab with the anti-PD-L1 antibody atezolizumab significantly increased survival outcomes over sorafenib in the randomized phase III IMBRAVE150 trial.^22^ Atezolizumab/bevacizumab objective response rate of 27.3%, including a 5.5% complete response rate and many durable responses, stands as a breakthrough in advanced HCC treatment. Whether anti-VEGFR TKIs—that also target vascular pericytes—can also reprogram the immunosuppressive microenvironment of HCC and enhance ICB efficacy in a similar manner remains unknown. Furthermore, whether combinatorial approaches using multitargeted anti-VEGFR TKIs could address treatment resistance via targeting alternative mechanisms remains unknown. Of note, the initial clinical experience with combination of lenvatinib with the anti-PD1 antibody pembrolizumab showed high response rate and led to a randomized phase III trial. Moreover, a recent phase Ib dose-escalation study evaluated the efficacy of regorafenib at 80–160 mg/day and anti-PD-1 therapy (nivolumab) in refractory gastric or colon cancer. After reducing the regorafenib dose to 80–120 mg/day during the dose-expansion part, study results showed an intriguing objective response rates of 40% overall, including a 36% response rate in microsatellite stable metastatic colorectal cancer, a notoriously ‘immunologically cold’ ICB-resistant tumor.^23^

Here, we studied the dose-dependent impact of regorafenib, which directly targets VEGFRs, Tie2 and PDGFR but may also have indirect inhibitory activity against signal transducer and activator of transcription 3 (STAT3), an immune-relevant target, in models of HCC with underlying liver damage.^24 25^

## Methods

### Cells

We used two murine HCC cell lines in this study: RIL-175 (a *p53/Hras* mutant line syngeneic to C57Bl/6 mouse strain background),^26^ kindly provided by Dr Tim Greten (NIH), and HCA-1, a line derived from a spontaneous HCC in a C3H mouse established in our laboratory.^27 28^

### Mouse models of orthotopic HCC and liver damage

For orthotopic tumor generation, RIL-175 cells were implanted in male mice of C57Bl/6 strain background (wild-type, *Rag1*^–/–^, *Cxcr3*^–/–^, Jackson Labs) and HCA-1 cells in male C3H mice.^29 30^ For spontaneous HCC generation, we used *Mst1*^–/–^*Mst2*^f/–^ mice (both genders).

### Agents and treatments

Regorafenib was provided by Bayer and administered by oral gavage daily at a dose of 5, 10 and 20 mg/kg in 34% 1,2-propandiol (Sigma-Aldrich), 34% PEG400 (Sigma-Aldrich), 12% pluronic F68 (Thermo Fischer Scientific, Waltham, Massachusetts, USA), and 20% water, as per manufacturer’s recommendation. Mouse anti-PD1 antibody (clone RMP-014) was purchased from BioXcell (Lebanon, New Hampshire, USA) and Mouse IgG isotype control was purchased from Thermo Fischer Scientific. The STAT3 inhibitor LLL12 was purchased from BioVision (Milpitas, California, USA). Antimouse PD1 antibody or IgG (control) were given intraperitoneally at a dose of 10 mg/kg thrice weekly, as described.^11^ Daily gavage of vehicle and intraperitoneal injection of 10 mg/kg of isotype-matched IgG were given as control treatments for anti-PD1 antibody. For the survival studies, moribund status was used as the end point and moribund was defined as symptoms of prolonged distress, >15% of weight loss compared with the starting date, body condition score >2 and tumor size of >24 mm in diameter.

### Patient samples

Formalin-fixed paraffin-embedded HCC tissue and blood samples were obtained with informed consent from a published cohort of deidentified patients who underwent liver resection at the department of Surgery at Fundeni Clinical Institute, Bucharest, Romania.^31^ Furthermore, we collected blood samples with informed consent from patients treated by regorafenib as standard of care (HCC) or in a phase I clinical trial of regorafenib monotherapy in patients with acute myeloid leukemia (AML). Peripheral blood was collected before regorafenib therapy and after 2 weeks of treatment and processed in a Clinical Laboratory Improvement Amendments-certified facility of the Steele Laboratories at MGH.

### Flow cytometry analysis

Prior to immunostaining, cells were washed with the buffer and fixed and permeabilized with FoxP3/Transcription Factor Staining Buffer Set (eBioscience/Thermo Fischer Scientific) to stain the intracellular markers. Harvested cells were incubated in Dulbecco's Modified Eagle Medium with cell activation cocktail with brefeldin A (Biolegend) for 4 hour at 37°C. The cells were stained with the antibodies of cell surface and intracellular marker in the buffer with brefeldin A.

Antimouse CD16/32 antibody (clone 93, Biolegend, San Diego, California, USA) was added for FcR blockade, and incubated for 5 min at room temperature. After another washing step, antibodies for cell phenotyping were added, and cells were incubated for 40 min at room temperature. The monoclonal antibodies used for flow cytometry analysis were for: CD45 (30-F11), CD3e (145-2 C11), CD4 (RM4-5), CD8a (5H10-1), Foxp3 (FJK-16s), CD39 (Duha59), CXCR3 (CXCR3-173), CD11b (M1/70), Ly6C (HK1.4), Ly6G, CD206 (C068C2), MHC-II (M5/114.15.2), interferon gamma (IFN-γ) (XMG1.2), PD1 (29F.1A12) (eBioscience).

### Immunohistochemistry

For analyses of endothelial and perivascular cells, tumor tissues containing the total areas of CD31 (clone DIA-310, Dianova, Germany) positive endothelial cells and α-smooth muscle actin (α-SMA) (Sigma-Aldrich, Saint Louis, Missouri, USA) positive pericytes were identified by scanning tumor sections under ×10 magnification and were counted in five random fields under ×200 magnification. CD8+ lymphocytes were detected using anti-CD8b antibody (eBioscience). Five random fields were selected from the tumor center, which did not include the edge of the tumor. Hypoxia was identified and quantified using immunostaining for anticarbonic anhydrase (CA)-IX (rabbit monoclonal; dilution, 1:100, Abcam). These data were analyzed using ImageJ (US NIH) and Photoshop (Adobe Systems, San Jose, California, USA) software. Analysis was performed using a laser-scanning confocal microscope (Olympus, FV-1000). Anticleaved caspase-3 antibody (Abcam) was used to assess the cell apoptosis in the tumor tissues, and antiphospho-STAT3 antibody (Abcam) for quantitative assessment of p-STAT3. For the latter, immunostained whole slide tumor sections were scanned on a Hamamatsu NanoZoomer S360 slide scanner. For quantification purpose, whole images were analyzed for at least three representative areas of vital tumor which was defined as region-of-interest.

### RNA in situ hybridization (RNA-Scope)

The RNA-Scope assay was performed according to manufacturer’s instructions.^32^ Briefly, formaldehyde-fixed and paraffin-embedded human and mouse HCC tissue blocks were cut in 5 µm sections, pretreated for 30 min, following digestion and hybridization at 40°C in a HybEZ oven with both dual labeling probes antimurine CXCR3 probe (Mm-CXCR3; 402511-C2) and CXCL10 (Mm-CXCL10; 408921) or human CXCL10 (Hs-CXCL10; 311851), all from Advanced Cell Diagnostics. Counterstaining was performed using hematoxylin for 10 s.

### Pharmacokinetic analysis of regorafenib and metabolites

From the orthotopic HCC and liver damage mouse models described above, blood samples were collected at 1, 4, 7 and 24 hours after once-daily oral administration of 5, 10 and 20 mg/kg regorafenib, respectively, for 5 days. Anti-PD1 antibody was given on days 1 and 4 with dose of 10 mg/kg by intraperitoneal injection. Blood samples were drawn from four to five animals per sampling time point. Regorafenib and its active metabolites M-2, M-4 and M-5 were analyzed in plasma samples using a validated bioanalytical liquid chromatography-tandem mass spectrometry.^25 33^ Pharmacokinetic (PK) parameters were derived from the area under the plasma concentration versus time curve (AUC) using a non-compartmental analysis.

### Statistical analysis

All statistical analyzes were performed using Stata software, V.14.1 (StataCorp, College Station, Texas, USA). Error bars indicate the SEM. Differences were considered significant when p values were <0.05. Quantitative variables were compared using Student’s t-test. Analysis of experiments with more than two groups were performed using one-way analysis of variance with Scheffe’s correction for multiple comparisons. Wilcoxon signed-rank test was used for analysis of marker changes over time. Based on tumor growth delay data, we estimated that an n>10 mice/group were required to ensure an alpha level of 5% (two-sided) and beta level of 20% (two-sided) for the survival analysis. Median overall survival was estimated using the Kaplan-Meier method. Statistical analyses in the survival experiments were performed by Cox proportional hazard model and HR and 95% CIs were calculated as well.

See additional materials and methods in online supplemental material.

10.1136/jitc-2020-001435.supp1 Supplementary data

## Results

### PD1 blockade combined with an intermediate dose of regorafenib induces vessel normalization and increases regorafenib delivery and activity

**Figure 1:**
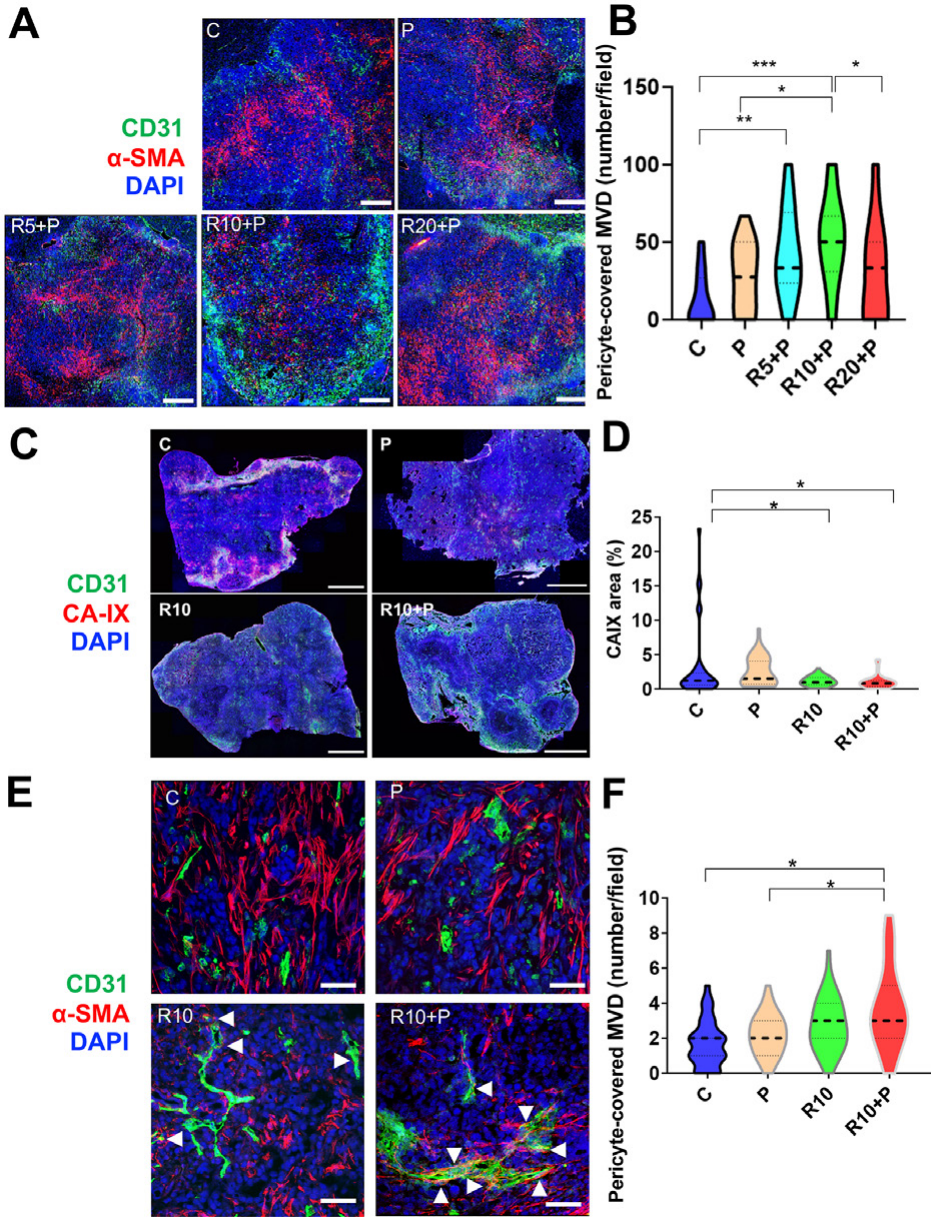
Dose-dependent effects of regorafenib on vascular normalization when used in combination with programmed cell death protein 1 (PD1) blockade in RIL-175 hepatocellular carcinoma (HCC) model. (A, B) Mice with established (4–5 mm in diameter) HCCs were treated with anti-PD1 therapy (P) alone or in combination with 5 mg/kg (R5), 10 mg/kg (R10) or 20 mg/kg (R20) regorafenib versus control (C) (n=8 mice per group, 1-week treatment). We used immunofluorescence (IF) for evaluation of tumor vessels by CD31 and α-smooth muscle actin (α-SMA) immunostaining. Quantitative analysis of IF data showed increased pericyte coverage only after R10+P treatment (plots in B). Representative IF of tumor sections after staining for CD31 (for endothelial cells in green) and α-SMA (for pericytes in red) and DAPI counterstaining (in blue). Five random fields from the tumor center (defined as no inclusion of the edge of the tumor) were evaluated for each sample. Data represent mean values. Scale bars=500 µm. (C) Representative IF confocal microscopy images of tumor vessels using and CD31 staining (green) and tissue hypoxia using CA-IX staining (red) of whole liver sections; scale bars=2 mm. (D) Tissue hypoxia was significantly reduced by 10 mg/kg regorafenib treatment alone or with anti-PD1 blockade (n=5 mice, 12 days of treatment, 5 images/tumor sample). (E) Representative IF confocal microscopy images of mature tumor vessels using CD31 staining (green) and α-SMA staining (red) of RIL-175 tumors; scale bars, 100 µm. (F) CD31+α-SMA+ mature vessel density was significantly increased in the combination treatment group (n=5 mice, 12 days of treatment, images/tumor sample). Data represent mean±SEM. *P<0.05; **p<0.01; ***p<0.001.

We first evaluated the dose-dependent effects of regorafenib in combination with anti-PD1 antibodies on HCC vessels in a time-matched manner (after 1 week of treatment). CD31+ surface area—a measure of microvascular density (MVD)—was increased after treatment with 10 mg/kg regorafenib and anti-PD1 antibody compared with all the other groups in RIL-175 model (online supplemental figure S1A). Moreover, pericyte coverage was significantly increased, and tissue hypoxia showed a tendency for decrease, after treatment with anti-PD1 antibody with 10 mg/kg but not with 20 mg/kg of regorafenib (figure 1A, B and online supplemental figure S1B, C).

10.1136/jitc-2020-001435.supp2 Supplementary data

To further understand the effects of the intermediate dose (10 mg/kg) of regorafenib, we examined its effects alone or in combination with anti-PD1 treatment at a later time-point in the same model (day 12). We found that the significant increase in MVD after 10 mg/kg regorafenib alone and with anti-PD1 treatment compared with control was maintained at this time-point (online supplemental figure S1B). Moreover, the hypoxic tissue area, measured by CA-IX staining, was significantly decreased in the intermediate dose regorafenib and combination treatment groups compared with control (figure 1C, D and online supplemental figure S1D, E). Furthermore, the mature (pericyte-covered) MVD was significantly increased only in the combination group, consistent with both structural and functional vascular normalization in HCC after regorafenib at the 10 mg/kg dose with PD1 blockade (figure 1E, F).

In addition, we evaluated PK parameters in HCC-bearing mice after regorafenib administration alone at doses of 5, 10 and 20 mg/kg or in combination with anti-PD1 therapy at the 10 mg/kg regorafenib dose for 5 days to achieve a steady state. We found differential plasma versus tumor PK for regorafenib and anti-PD1 therapy. In plasma, the PK parameters area under the curve within the observation period of 24 hours (AUC_0–24_) and maximum/peak concentration (C_max_) showed a dose-proportional increased for regorafenib (online supplemental table S1). However, while tumor AUC_0–24_ and C_max_ also showed a dose-proportional increase for regorafenib, addition of anti-PD1 therapy to regorafenib 10 mg/kg further increased tumor exposure of the drug (online supplemental table S1 and online supplemental figure S1F, G). Consistent with these data, we found an increase in the biologically active metabolite of regorafenib M-4, which occurs prominently in mice, from the lower to higher dose group. While plasma PK of M-4 were not changed by addition of anti-PD1 antibody to regorafenib 10 mg/kg, tumor PK parameters also showed trends for increased M-4 (online supplemental table S1). The concentrations of the active metabolites M-2 and M-5 were below the detection limit of the assay in most samples. Collectively, these results show that an intermediate dose of regorafenib with anti-PD1 blockade is required to promote vascular normalization, which reduces hypoxia and increases regorafenib delivery and activity in the HCC tissue.

### Regorafenib at 10 mg/kg dose is required to enhance CTL infiltration and activation when used in combination with PD1 blockade

**Figure 2:**
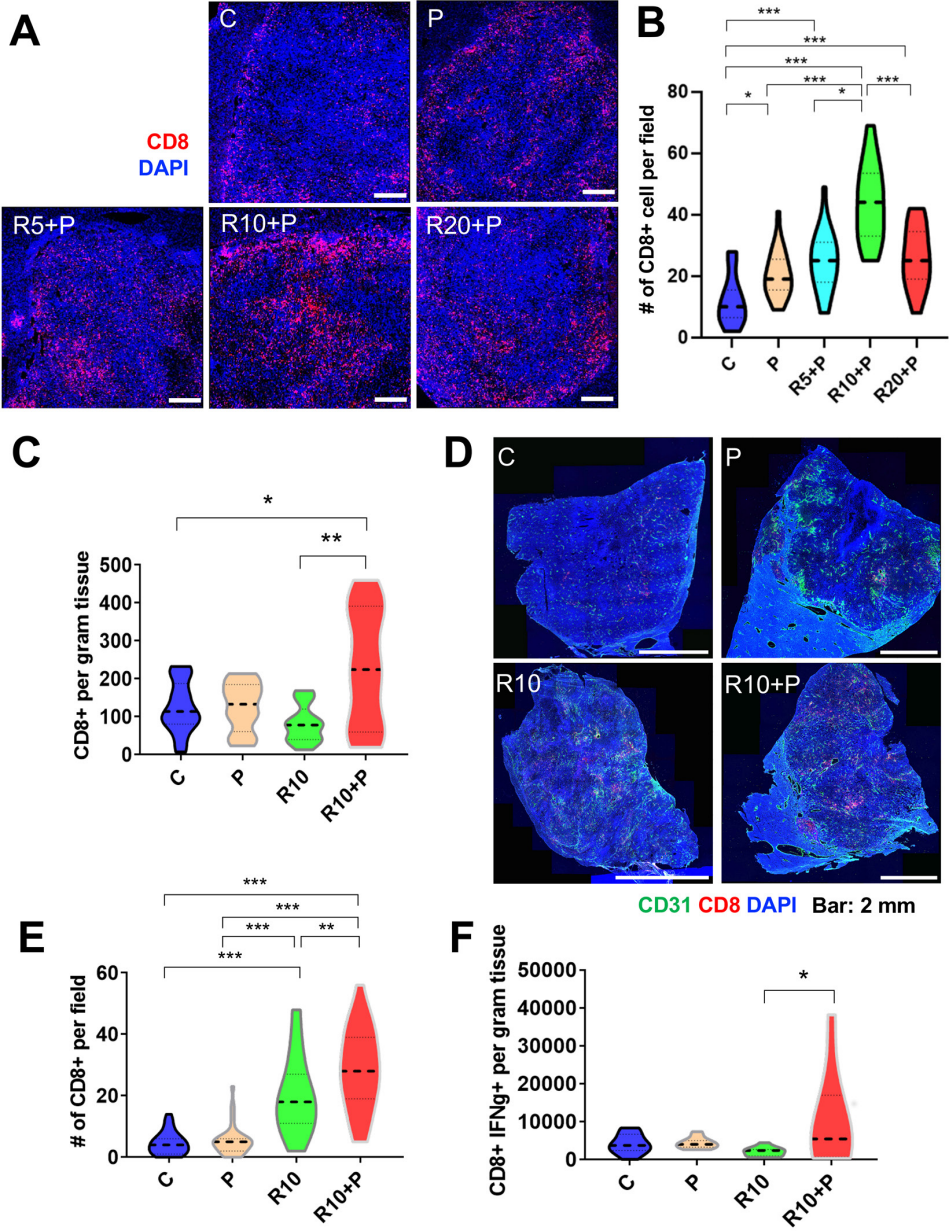
Intermediate dose of regorafenib with antiprogrammed cell death protein 1 (anti-PD1) therapy increases intratumoral CD8+ cytotoxic T lymphocyte (CTL) infiltration and activation in RIL-175 model. (A) Mice with established (4–5 mm in diameter) hepatocellular carcinomas (HCCs) were treated with anti-PD1 therapy (P) alone or in combination with 5 mg/kg (R5), 10 mg/kg (R10) or 20 mg/kg (R20) regorafenib versus control (C) (n=8 mice per group, 1 week treatment). We used immunofluorescence (IF) for evaluation of T-cell infiltration by CD8 immunostaining. Representative IF of tumor sections after staining for CD8 (for T cells in red) and DAPI counterstaining (in blue). Five random fields from the tumor center (defined as no inclusion of the edge of the tumor) were evaluated for each sample. Data represent mean values. Scale bars=500 µm. (B) Quantitative analysis of IF data showed a significant increase in CD8+ T cells after R10+ P treatment compared with all other groups. (C) Regorafenib (10 mg/kg daily, R10) combined with anti-PD-1 therapy (P) significantly increased the number of CD8+ T cells per gram RIL-175 tumor tissue measured by flow cytometry versus control or R10-treated mice (n=11–13 mice, 12 days of treatment). (D) Representative IF confocal microscopy images of CD8 T-cell distribution (in red) in whole RIL-175 tumor tissue sections; CD31 staining of vessels in green and DAPI counterstaining in blue; scale bars=2 mm. (E) Quantification of intratumoral CTL infiltration in RIL-175 HCCs showing a more substantial increase in intratumoral CTL infiltration in R10/P-treated group, which was significantly higher than each treatment alone or control (n=5 mice, 12 days of treatment, 5 random fields from the tumor center (defined as no inclusion of the edge of the tumor) per sample). (F) R10/P combination therapy significantly increased the number of CD8+ interferon gamma (IFN-γ)+CTLs per gram of RIL-175 tumor tissue measured by flow cytometry versus each treatment alone or control (n=10–13 samples, 12 days of treatment). *P<0.05; **p<0.01; ***p<0.001.

Having demonstrated that regorafenib given at 10 mg/kg dose with anti-PD1 antibody normalized tumor vessels’ structure and function, we next investigated the effects of this combination therapy on antitumor immunity in HCC models. First, we evaluated the intratumoral infiltration and distribution of CD8 T cells in RIL-175 tumors. The total number of CD8 T-cells infiltrating inside the tumor, evaluated by IF, was significantly increased after 1 week of treatment in the tumors from mice receiving anti-PD1 therapy, alone or with regorafenib at 5, 10 or 20 mg/kg. However, the CD8 T cell infiltration inside the tumor was significantly higher after 10 mg/kg regorafenib and anti-PD1 antibody compared with all other groups (figure 2A, B).

In separate experiments, we tested whether this effect of regorafenib 10 mg/kg/anti-PD1 therapy was maintained at a later time-point (day 12 of treatment). We found that the total number of CD8 T cells measured by flow cytometry in tissue was significantly increased in the combination therapy group as compared with all the other treatment groups (figure 2C), despite the persistence of immunosuppressive myeloid cells (online supplemental figure S2A, B). Furthermore, when we evaluated the intratumoral distribution of the CD8 T cells by IF, we found that their infiltration was significantly increased in the combination group versus other treatment groups (figure 2D, E and online supplemental figure S2C). In addition, combination therapy promoted the activation of CD8 T cells, as measured by IFN-γ expression. Using western blot analysis, we found that overall tissue IFN-γ expression was increased after combination therapy (online supplemental figure S2D); flow cytometry validated that CD8+IFN-γ+activated CTLs were significantly increased only in the combination therapy group (figure 2F).

### Regorafenib increases CXCL10 production by HCC cells

**Figure 3:**
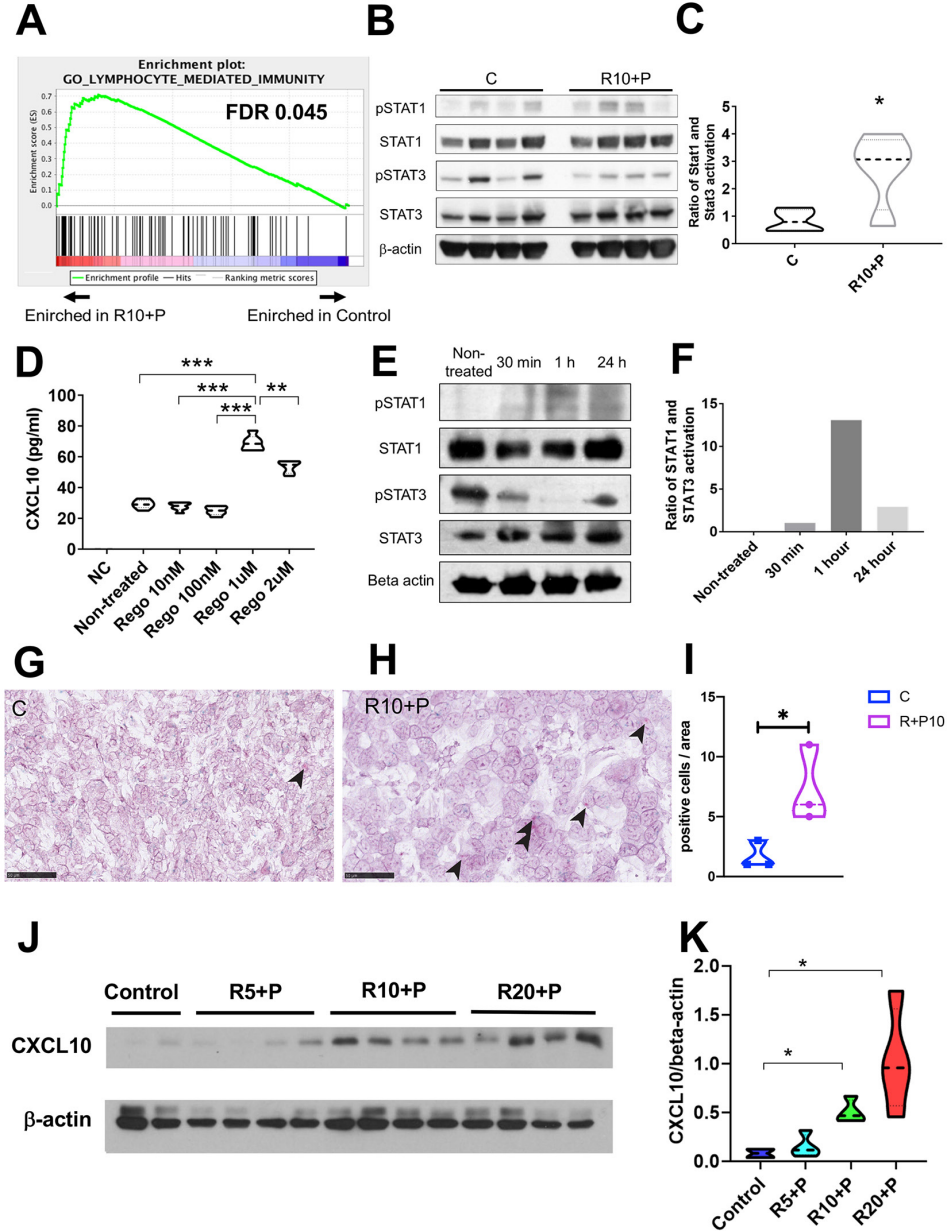
Combined treatment with regorafenib with antiprogrammed cell death protein 1 (anti-PD1) antibodies inhibits signal transducer and activator of transcription (STAT) activation and increases CXCL10 production by hepatocellular carcinoma (HCC) cells. (A) Changes induced by combination of regorafenib (10 mg/kg) with anti-PD1 antibody in immune-related pathways from the gene set enrichment analysis (GSEA) using curated gene sets in RIL-175 HCC model (see also online supplemental dataset S1 and figure S2E). (B, C) Changes induced by combination of regorafenib (10 mg/kg) with anti-PD1 antibody in STAT3 and STAT1 activity (B), showing an increased ratio of phosphorylated (p)-STAT1/p-STAT3 (normalized to total STAT1 and STAT3, respectively) by western blot analysis of whole RIL-175 HCC tissue (C) (n=4 mice). (D) Treatment with regorafenib increases CXCL10 expression by RIL-175 HCC cells in vitro after 36 hours, in a dose-dependent manner (n=6). Culture medium alone was used as a negative control (NC). (E, F) Changes induced by regorafenib (1 µM) in STAT3 and STAT1 activity (E), showing an increased ratio of p-STAT1/p-STAT3 (normalized to total STAT1 and STAT3, respectively) by western blot analysis of RIL-175 HCC cells (F). (G, H) Representative in situ hybridization (ISH) pictures of CXCL10 (red dots, arrow heads) and CXCR3 (green dots) on RIL-175 HCC tissue from the control group (G) versus combination therapy (RP) group (H). Scale bars=50 µm (G, H). (I) Combination therapy significantly increases in the number of HCC cells expressing CXCL10 compared with control. Data represent mean±SEM. (J, K) Combination therapy increases CXCL10 protein expression in whole RIL-175 tumor tissue 6 hours and 12 hours after in vivo when regorafenib treatment was used at doses of 10 mg/kg (R10) and 20 mg/kg (R20) (n=3) by western blot analysis (J); quantitative data of CXCL10/β-actin ratios are shown in (K). C, control; P, anti-PD1 antibody; R, regorafenib 10 mg/kg; R10-P, regorafenib 10 mg/kg+anti-PD1 antibody; n=3–4 per group. *P<0.05; **p<0.01; ***p<0.001.

To investigate the mechanism mediating the increased intratumoral CTL infiltration after combined regorafenib 10 mg/kg and anti-PD1 treatment in HCC, we examined the transcriptional changes in whole tumor lysate using RNA sequencing analysis after 1 week of treatment (online supplemental dataset S1 and figure S2E). Gene set enrichment analysis (GSEA) using curated gene sets (Gene Ontology) showed significant changes in multiple pathways, including those related to immune response and factors related to CTL trafficking in RIL-175 tumor tissues (figure 3A). We found that an unbiased GSEA using a previously published ‘poor prognostic angiogenic gene’ signature^20^ showed that these genes were enriched in the control group compared with regorafenib (10 mg/kg) plus anti-PD1 therapy (online supplemental figure S2F). These data further support the vascular normalization effect which was seen in the time-matched study presented in figure 1.

On the other hand, we found significant upregulation in the gene expression of both the *Cxcr3* receptor and its ligand *Cxcl10* after combination treatment (compared with control) among the top hits (online supplemental table S2). In addition, we measured protein concentration in the whole tumor tissue (used in the PK experiments) by ELISA and found that CXCL10 showed a non-significant trend for increase after treatment with intermediate dose regorafenib and anti-PD1 antibody (n=4) (online supplemental figure S3A). Of note, we have previously reported RNA sequencing analysis after dual antibody blockade of VEGFR2 and PD-1 antibody using the same orthotopic HCC model, but found no difference in CXCL10 or CXCR3 expression.^21^ These data indicate that upregulation of the CXCL10/CXCR3 axis may be due to the multitargeted (VEGFR-independent) activity of regorafenib.

In the same samples used for the PK study, we also found that regorafenib treatment inhibited phosphorylated (p)STAT3 levels in HCC tissues (online supplemental figure S3B, C). Thus, we evaluated the pSTAT1/pSTAT3 ratio after combination of regorafenib (10 mg/kg)/anti-PD1 treatment. We found an increase in the ratio of STAT1/STAT3 phosphorylation levels after combination treatment (figure 3B, C). These results indicate that regorafenib treatment can shift the ratio between activated STAT1 and STAT3, potentially leading to increased CXCL10 production. We also used the TCGA database to confirm the correlation between the *CXCL10* expression and the other immune-related genes. We found that the *CXCR3, STAT1, CD8A* and *IFNG* genes were positively correlated in human HCC tissue (online supplemental figure S3D).

To determine if CXCL10 was produced by murine HCC cells in response to regorafenib treatment, we next cultured RIL-175 cells with various concentration of regorafenib in vitro and measured the CXCL10 levels by ELISA. Regorafenib treatment significantly increased the level of CXCL10 secreted by RIL-175 cells and to a lesser extent in HCA1 cells, with a peak concentration at 1 µM (figure 3D and online supplemental figure S3E). Moreover, we also found that a selective and direct STAT3 inhibitor (LLL12) significantly increased CXCL10 expression in RIL-175 cells (online supplemental figure S3F). Furthermore, we confirmed that the STAT1 activation and STAT3 inhibition were also seen in the RIL-175 cells culture with 1 µM of regorafenib in vitro (figure 3E, F).

To validate whether regorafenib treatment can increase expression of CXCL10 in vivo, and to identify a cell population in HCC expressing CXCL10 and CXCR3, we performed an RNA-Scope in situ hybridization (ISH) dual labeling for both markers. In line with the in vitro findings, we found an increased level of CXCL10 mRNA in the combination group of regorafenib and anti-PD1 antibody in the murine HCC tissue (figure 3G–I). In addition, dual labeling of both CXCL10 and CXCR3 confirmed that TILs in proximity of CXCL10-expressing HCC cells showed expression of CXCR3 mRNA. CXCL10 expression was almost exclusively detected in cancer cells, based on histological analysis performed by a trained liver pathologist (AQ). Moreover, flow cytometric analyses confirmed the increased levels of CXCR3+CD8+ T cells after combined regorafenib/anti-PD1 therapy in the tumor tissue but not in the spleen (online supplemental figure S3G, H).

Finally, we measured the dose-dependent changes in intratumoral CXCL10 protein expression after regorafenib treatment in mice. We found that regorafenib treatment alone at 10 mg/kg and 20 mg/kg increased protein levels of CXCL10 evaluated in whole tumor tissue lysate by western blot analysis as early as 6 hours after treatment (online supplemental figure S3I, J). Moreover, when we evaluated CXCL10 expression after prolonged combination treatment with regorafenib and anti-PD1 antibodies (for 1 week in the PK studies), we found increased protein expression level of CXCL10 for the intermediate (10 mg/kg) and the high dose (20 mg/kg) of regorafenib but not for the low dose (5 mg/kg) by western blot analysis in whole tumor tissue lysate (figure 3J, K).

Collectively, these results indicate that CXCL10 expression by HCC cells is increased after regorafenib treatment due to inhibition of STAT3 activity leading to increased intratumoral infiltration CD8+CXCR3+ T cells.

### Survival benefit of regorafenib combined with anti-PD1 treatment is regorafenib dose-dependent

**Figure 4:**
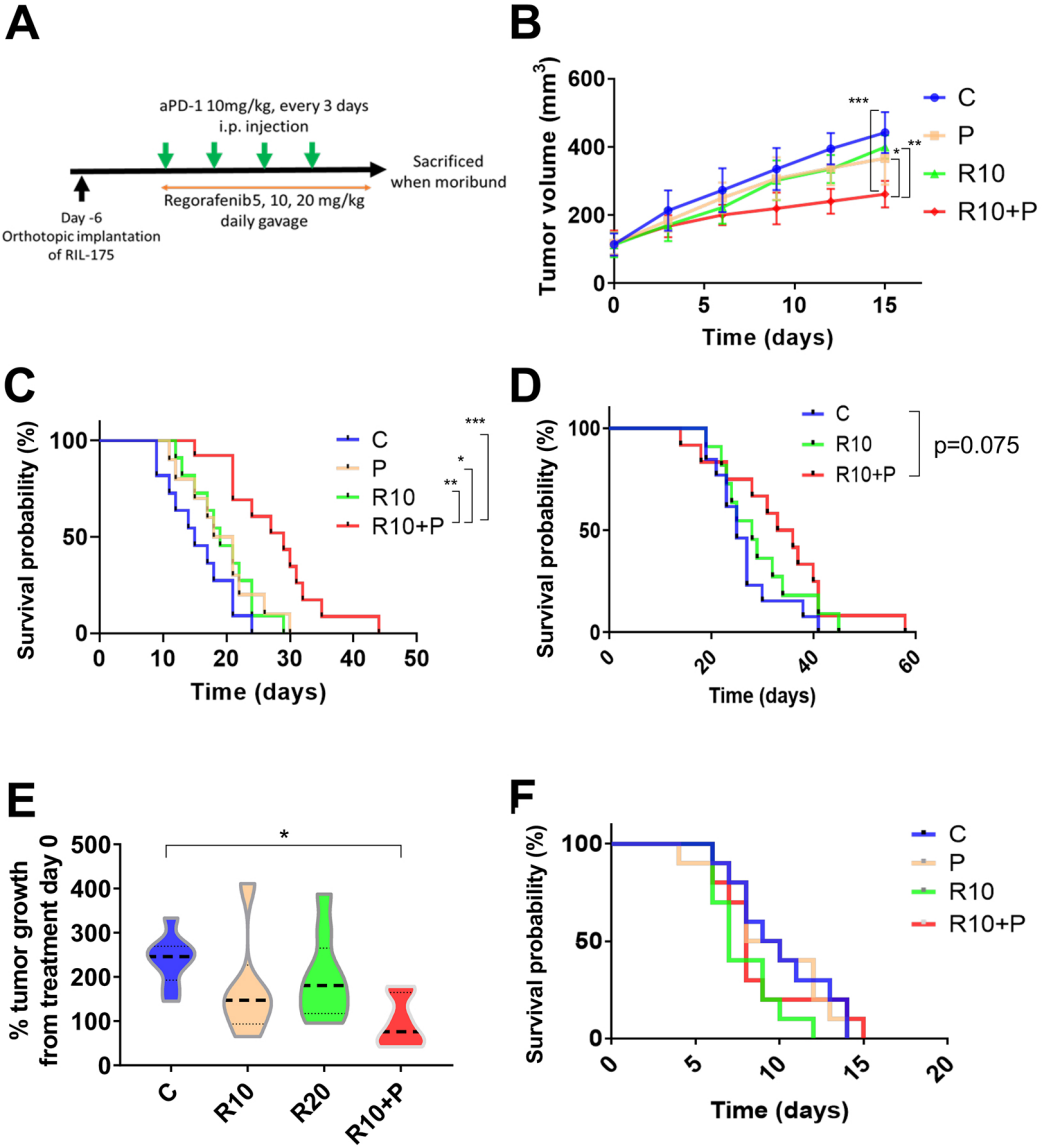
Intermediate dose of regorafenib shows efficacy when combined with antiprogrammed cell death protein 1 (anti-PD1) blockade in the RIL-175 mouse model of advanced hepatocellular carcinoma (HCC). (A) Example of tumor implantation and treatment schedule for survival studies in HCC models. (B, C) Survival experiment data from RIL-175 orthotopic mouse model. Tumor growth was significantly delayed in the group treated with a combination of regorafenib 10 mg/kg and anti-PD1 antibody (aPD1) compared with the other groups (n=10–13 mice) (B); moreover, this combination signiﬁcantly prolonged overall survival (C). (D) Survival experiment data from HCA-1 highly metastatic orthotopic mouse model. Mice treated with combination with R10+P show a non-significant trend for increased survival versus control (HR=0.48, p=0.075) (n=11–13 mice). (E) Tumor growth is delayed after combination of R10+P in a spontaneous HCC model using *Mst1*^–/–^*Mst2*^F/–^ mice (n=6–9 mice). (F) Treatment with regorafenib (10 mg/kg daily, R10) alone or in combination with anti-PD-1 therapy shows no benefit in survival (Kaplan-Meier survival distributions) compared with control. *P<0.05, **p<0.01, ***p<0.001. C, control; P, anti-PD1; R10, regorafenib 10 mg/kg daily; R10+P, regorafenib 10 mg/kg+anti-PD1. Error bars, SEM.

We finally tested the efficacy of combining anti-PD1 antibody with regorafenib dose versus each agent alone or control in three different mouse models (orthotopic RIL-175 and HCA-1 grafts, *Mst1/Mst2*-KO model) with underlying liver damage (figure 4A). In the RIL-175 HCC model, we found that combination treatment showed tumor growth delay but only when regorafenib was used at the dose of 10 mg/kg with anti-PD1 therapy (figure 4B). Moreover, regorafenib (10 mg/kg) plus anti-PD1 group showed a significant and substantial survival benefit (median survival of 29 days, more than double compared control group in this model) with an HR (HR=0.17; p<0.001) (figure 4C). The low dose (5 mg/kg) of regorafenib plus anti-PD1 therapy showed no significant growth delay and only a non-significant trend for survival benefit (HR=0.47; p=0.089) (online supplemental figure S4A). When we compared the efficacy of the combination treatment of anti-PD1 therapy with regorafenib at 10 mg/kg versus anti-PD1 therapy with a high dose (20 mg/kg) of regorafenib, we found that only regorafenib at 10 mg/kg significantly improved survival compared with control group (HR=0.24; p=0.004) (online supplemental figure S4B).

We also tested the efficacy of regorafenib combined with anti-PD1 treatment in highly metastatic HCC model with underlying liver damage (HCA-1 grafted in C3H mice). Similar to the RIL-175 model, we found a trend for increased survival for the combination when regorafenib was used at 10 mg/kg (figure 4D); of note, no activity was seen when we combined regorafenib at 20 mg/kg with anti-PD1 therapy (online supplemental figure S4C). Moreover, we tested anti-PD1 therapy with regorafenib at 10 mg/kg against spontaneous HCCs developed in *Mst1/Mst2-*deficient mice and found that it significantly delayed tumor growth (figure 4E). We did not detect any overt toxicity (eg, body weight loss) with any of the regimens tested, consistent with prior experience with this drug in mouse models (online supplemental figure S4D).^34^

Finally, when we repeated the survival experiment in an orthotopic RIL-175 model in *Rag1*^–/–^/C57Bl/6 mice, which lack functional T cells, we found no significant benefit for regorafenib 10 mg/kg with or without anti-PD1 antibody (figure 4F).

These results show the safety and efficacy of combining regorafenib at 10 mg/kg with PD1 blockade in mice, and the requirement of functional T cells for the antitumor effects of this combination therapy.

### Combination therapy of regorafenib with anti-PD1 antibody promotes CTL infiltration via CXCL10/CXCR3 axis

**Figure 5:**
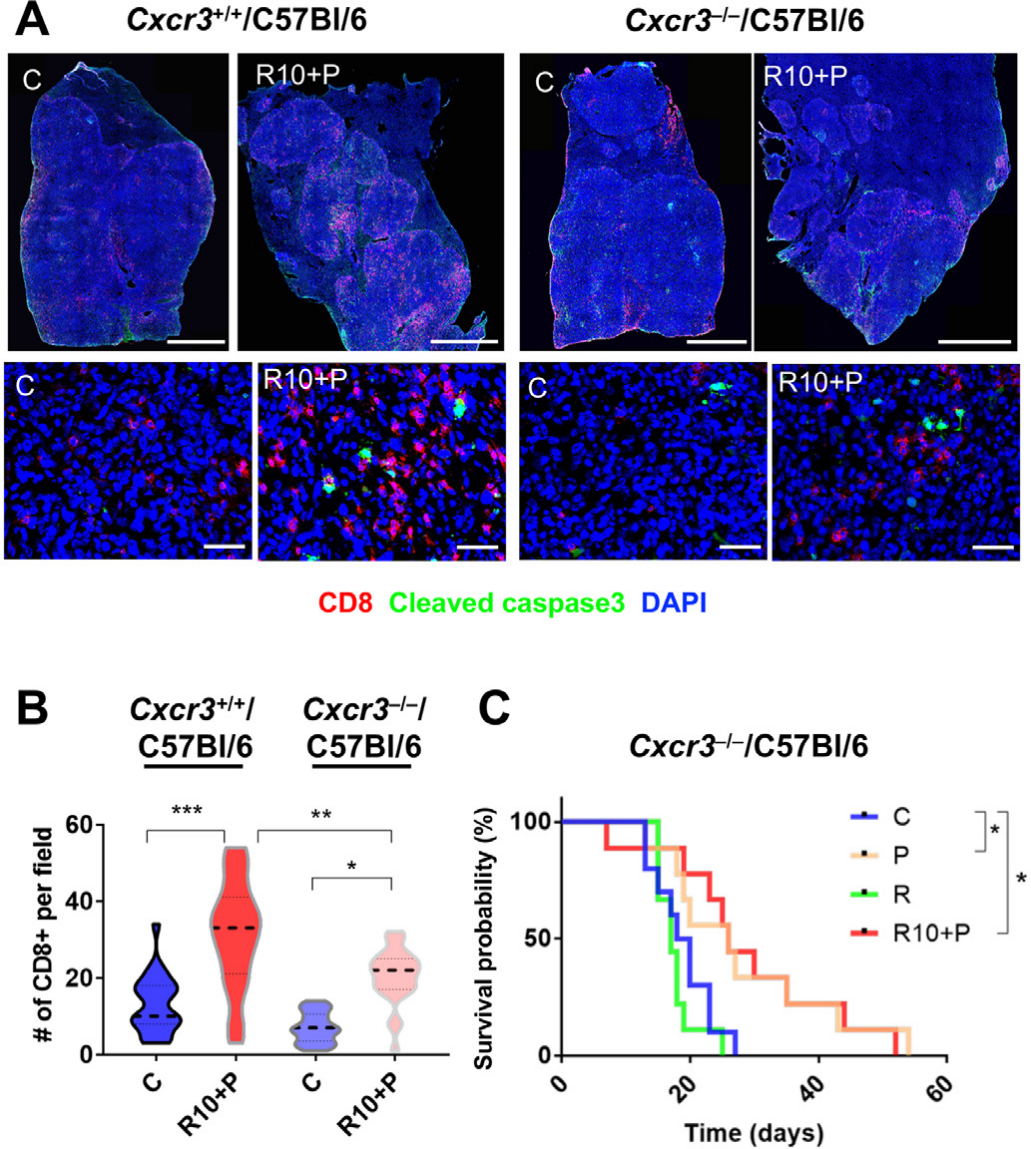
Increased intratumoral infiltration by CD8+ cytotoxic T lymphocytes (CTLs) after regorafenib plus antiprogrammed cell death protein 1 (anti-PD1) treatment is mediated by CXCL10/CXCR3 axis. (A, B) Representative immunofluorescence (IF) confocal microscopy images of whole liver sections (upper panel, low magnification, scale bars=2 mm; lower panel, high magnification, scale bars=50 µm) showing increased intratumoral localization of CD8+ CTLs co-localized with areas of cell apoptosis in orthotopic RIL-175 tumors in *Cxcr3*^+/+^/C57Bl/6 mice (upper panel) compared with orthotopic tumors grown in *Cxcr3*^–/–^/C57Bl/6 mice (lower panel) (A) after 1 week of regorafenib/anti-PD1 combination treatment. (B) Quantification of IF data showing that intratumoral CTL infiltration after regorafenib/anti-PD1 combination treatment is prevented when tumors are grown in *Cxcr3*^–/–^/C57Bl/6 mice (n=5 samples, 5 random areas/samples): C, control; R10+P, regorafenib 10 mg/kg+anti-PD1 antibody. (C) The added survival benefit of regorafenib with anti-PD1 blockade over anti-PD1 therapy alone is compromised in *Cxcr3*^–/–^/C57Bl/6 mice bearing hepatocellular carcinoma (HCC) (n=9–10 mice). C, control; P, anti-PD1 antibody; R10, regorafenib 10 mg/kg; R10+P, regorafenib 10 mg/kg+anti-PD1 antibody. Data represent mean±SEM. *P<0.05; **p<0.01; ***p<0.001.

To demonstrate that intratumoral CD8 T-cell infiltration after combination therapy of regorafenib with anti-PD1 antibody was mediated by CXCL10/CXCR3 axis, we next evaluated the effects of combined regorafenib (10 mg/kg)/anti-PD1 antibody therapy on RIL-175 HCC growing in *Cxcr3*-deficient (*Cxcr3*^–/–^) versus *Cxcr3*-proficient (*Cxcr3*^+/+^) C57Bl/6 mice. In time-matched experiments, we found that the number of CTL infiltrating HCC tissue was significantly decreased in *Cxcr3*^–/–^ mice compared with *Cxcr3*^+/+^ mice after 1 week of combination treatment (figure 5A, B). Of note, despite this decrease, combination therapy induced a small increase in intratumoral CD8+ T cells even in *Cxcr3*^–/–^ mice, likely mediated by vascular normalization. Moreover, when we evaluated the efficacy of regorafenib at 10 mg/kg with anti-PD1 antibody treatment in *Cxcr3*^–/–^ mice in survival experiments, we found no additional survival benefit for this combination over anti-PD1 therapy alone (figure 5C); these data are in contrast to the benefits seen with regorafenib at 10 mg/kg with anti-PD1 antibody treatment in *Cxcr3*^+/+^ tumor-bearing mice (figure 4C).

These results demonstrate that CXCR3 is a mediator of intratumoral CD8 T-cell infiltration—along with vascular normalization—and is critical for the added survival benefit after combination of regorafenib at 10 mg/kg with anti-PD1 therapy in HCC models.

### CXCL10 is detectable in human HCC, is measurable in blood samples from patients with cancer and is a potential biomarker of regorafenib’s immuno-modulatory activity

**Figure 6:**
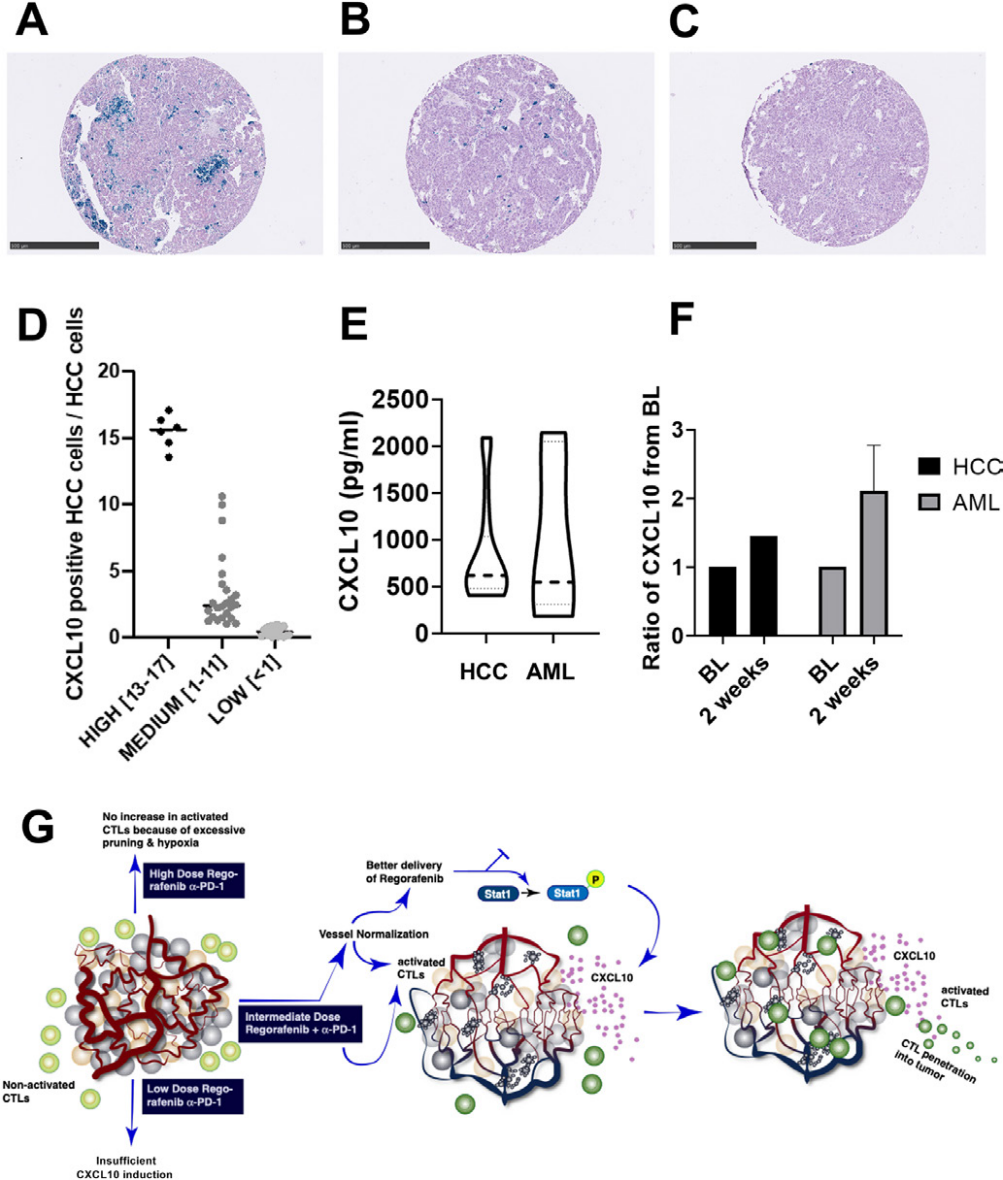
CXCL10 expression of in hepatocellular carcinoma (HCC) tissues and concentration in blood samples from patients with cancer. (A–C) CXCL10 expression by RNA-in situ hybridization (ISH) in human HCC samples; staining was scores ‘high’ (13%–17%) (A), medium (1%–11%) (B) or low (<1%) (C) fractions of HCC tumor cells with CXCL10 ISH positivity. Scale bars=500 µm. (D) Distribution of CXCL10 mRNA counts using ISH. (D) Concentration of CXCL10 in blood samples collected from HCC (n=20) and acute myeloid leukemia (AML) (n=8) patients prior to treatment. Detectable CXCL10 levels in all patients, with no significant difference between the two groups. (F) Increase of CXCL10 after 2 weeks of regorafenib treatment in patients with HCC (n=1) and AML (n=7). (G) Schematic diagram of the proposed mechanisms of benefit when using the appropriate dose of regorafenib combined with antiprogrammed cell death protein 1 (anti-PD1) therapy. Judicious dosing of regorafenib in combination with anti-PD1 antibodies can normalize HCC vessels, which results in increased drug delivery and synergy with antitumor immunity stimulated by concomitant anti-PD1 therapy. The increased CD8+ cytotoxic T lymphocyte (CTL) penetration and activation mediates this synergistic activity and is facilitated by CXCL10 expression by the cancer cells on inhibition of STAT3 phosphorylation by regorafenib.

We next performed RNA-ISH analysis of human tumor tissues and ELISA in blood samples from a published cohort of resectable patients with HCC.^31^ Furthermore, we collected blood samples from patients treated by regorafenib as standard of care (HCC); in addition, we examined the effect of regorafenib on blood CXCL10 levels in a clinical trial in a different disease—AML (NCT03042689). In line with preclinical data, we found that CXCL10 expression was detectable primarily in human HCC cells; >60% (30/48) untreated (surgically resected) tumors showed positivity for CXCL10 expression in HCC cells (figure 6A–D). Seven cases showed elevated levels of CXCL10 expression, with 13%–17% of tumor cells showing positivity for CXCL10 mRNA (figure 6A–D). Moreover, CXCL10 was detectable in the blood concentration of all patients with HCC (median: 617.9 pg/mL, n=20) and AML (median: 547.4 pg/mL, n=8). Finally, we found an increased concentration of plasma CXCL10 in both patients with advanced HCC (n=1) and AML (n=7) after 2 weeks of treatment (figure 6E, F). These data indicate that CXCL10 is objectively measurable and its concentration can increase in the blood of patients with HCC—supporting its future examination as a potential biomarker of the immunomodulatory activity of regorafenib treatment.

## Discussion/Conclusion

In this study, we demonstrate the impact of regorafenib dosing when combined with ICB on vascular structure and function, antitumor immunity, tumor growth and survival in HCC models in mice. A significant survival benefit was seen only in the group where PD1 blockade was combined with regorafenib at the intermediate regorafenib dose (10 mg/kg), which both normalized the vasculature and increased CXCL10 expression in HCC cells. Both effects were critical for achieving efficacy: use of a higher dose (20 mg/kg) of regorafenib in combination with anti-PD1 antibodies did not show a benefit despite increased regorafenib concentration and STAT3 inhibition as well as increased CXCL10 expression in the HCC tissue. This result is consistent with our published report using sorafenib and may be due to excessive pruning of vessels.^11^ On the other hand, use of a low dose of regorafenib (5 mg/kg) normalized the vessels but did not inhibit STAT3 activity or induce CXCL10 expression and failed to show a benefit when combined with anti-PD1 therapy. While the PK/PD of regorafenib is expected to be different in human patients with HCC, the parameters measured in HCC-bearing mice with liver damage provide proof-of-principle data on the relevance of judicious dosing of regorafenib to increase the efficacy of concomitant anti-PD1 immunotherapy (figure 6). These results are in line with predictions from mathematical models of dual VEGF/PD1 inhibition,^35^ as well as with emerging clinical data with combined use of lower dosed of regorafenib with anti-PD1 therapy in other gastrointestinal cancers.^23^

Moreover, we unraveled the pathophysiological, cellular and molecular mechanisms underlying the benefits achieved with the intermediate dose of regorafenib and anti-PD1 therapy. First, we found that combination therapy was more effective than regorafenib or anti-PD-1 therapy alone for normalizing the structure and the function of the abnormal HCC vasculature, that is, increased pericyte covered vessels, decreased hypoxia and increased drug uptake. This supports our recent finding that anti-PD1 therapy has vascular normalizing activity when combined with anti-VEGFR therapy in HCC.^21^ Second, using genetically engineered mouse models and transcriptional analyzes, we showed that the benefits of combined regorafenib/anti-PD1 therapy were mediated by T cells. We and others have shown that vascular normalization can enhance CD8 T-cell infiltration in tumors, including in HCC models.^16 21^ Here, we show that regorafenib can increase CXCL10 expression in cancer cells in vitro and in vivo, likely owing to its multitargeted activity. This effect further enhances intratumoral CXCR3+CD8+ T-cell infiltration in HCC. Through these two mechanisms, judicious dosing of regorafenib could enhance the benefits of ICB treatment in the ‘immunologically cold’ and ICB-resistant HCCs.

CXCL10 expression is stimulated by IFN-γ on downstream STAT1 activation.^36^ STAT1 and STAT3 have opposite effects in antitumor immune response. Previous studies have shown the reciprocal regulatory mechanism of STAT1 and STAT3 using the transgenic animals and concluded that their activation may be cross-regulated.^37 38^ Here, we found that intratumoral CD8 T-cell infiltration was promoted by CXCL10 upregulation in cancer cells after STAT3 inhibition by regorafenib and was facilitated in the context of PD1 blockade. STAT3 is a pathway known to promote cell survival, proliferation and immune tolerance,^37^ and may mediate cellular immune responses in HCC after multitargeted TKI treatment.^39^ Moreover, CXCR3 is expressed on a fraction of CTLs both in mouse and human HCC, as shown in our study and in the study by Zheng *et al*.^40^ A recent study demonstrated that intratumoral activity of the CXCR3 chemokine system is required for the efficacy of anti-PD1 therapy.^41^ Here, we show that CXCR3 expression in CTLs was critical for the survival benefit seen with regorafenib/anti-PD1 therapy, which was not seen in HCC-bearing mice deficient for CXCR3.

Previous studies have proposed blood circulating CXCL10 as a potential biomarker of response to pegylated IFN-antiviral treatment in patients with HCV, but its role in the migration of effector T cells to the infected liver remains unclear.^42^ We found that CXCL10 is often expressed by cancer cells in human HCC tissue specimens. We also evaluated the concentration of CXCL10 in blood of patients with HCC and AML and we found that the CXCL10 is detectable in all patients and is increased after regorafenib treatment.

Despite our use of three different murine HCC models to confirm the activity of regorafenib combined with anti-PD-1 antibody, clinical translation of our results will be challenging. Our models do not account for the significant heterogeneity seen with HCC in patients, and thus the effects of the regorafenib/anti-PD1 therapy will need to be studied across different subtypes and etiologies. Moreover, evaluation of CXCL10 and microenvironmental changes, and their role in patients with HCC, will need to be performed in larger studies. Such analyses would be most impactful if performed at the time of radiological response or resistance to regorafenib/anti-PD1 therapy. These limitations notwithstanding, these data provide clear evidence that CXCL10 is expressed by cancer cells and suggest that plasma CXCL10 changes should be further evaluated as a potential biomarker of regorafenib’s immunomodulatory effects, particularly in patients receiving concurrent immunotherapies.

In conclusion, we show here that regorafenib treatment can significantly enhance PD1 blockade effects in a dose-dependent manner in HCC models. The benefit was due to the activity of the two agents both on normalization of HCC vasculature and stimulation of anti-tumor immunity. Combination treatment inhibited STAT3 activity and increased the expression of CXCL10 chemokine, which increased both tumor penetration of activated CD8 T cells and survival. This concept is highly relevant for the future of development of combination treatment strategies in patients with advanced HCC using drugs (multitargeted TKIs or specific VEGFR and STAT3 inhibitors) with known pharmacology and toxicity profiles. Furthermore, the regorafenib-specific mechanism of action on tumor immune microenvironment is relevant in HCC treatment, and potentially in other cancers that metastasize to the liver.
