# Supplementary material for: Regorafenib combined with PD1 blockade increases CD8 T-cell infiltration by inducing CXCL10 expression in hepatocellular carcinoma
Source: J Immunother Cancer. 2020 Nov 24;8(2):e001435. doi: 10.1136/jitc-2020-001435 (PMC7689089; doi:10.1136/jitc-2020-001435)
Supplement: Supplementary data [file jitc-2020-001435supp001.pdf]

SUPPLEMENTARY MATERIAL

Materials and methods

Cell culture. Cell line authentication and mycoplasma contamination testing were performed prior to all experiments. RIL-175 cells were maintained in Dulbecco's modified medium (DMEM) with 20% fetal bovine serum (FBS) with pyruvic acid and 1% of penicillin/streptomycin. HCA-1 cells were maintained in DMEM with 10% FBS with pyruvic acid and 1% of penicillin/streptomycin.

Mouse models orthotopic HCC and liver damage. For orthotopic tumor generation, RIL-175 cells were implanted in male mice of C57Bl/6 strain background and HCA-1 cells in male C3H mice. One million HCC cells were suspended in an equal amount of PBS and Matrigel (Mediatech/Corning, Manassas, VA) and injected intrahepatically in 6-8-week-old male mice. For spontaneous HCC generation, we used *Mst1*$^{-/-}$*Mst2*$^{f/-}$ mice (both genders). In brief, Cre-adenoviruses at a concentration of 1x10^{7} pfu were injected via the tail vein at 5 weeks of age. For all models, liver damage was induced by oral administration of carbon tetrachloride (CCl4; Sigma-Aldrich, Saint Louis, MO) at a concentration of 20% in olive oil for 4-6 weeks, prior to tumor implantation and concomitantly with Cre-adenovirus injection in the genetically engineered *Mst1/2*-mutant model. All experimental procedures were performed at least twice after obtaining institutional approval.

Tumor growth was monitored using high-frequency ultrasonography every 3 days. Tumor volume was estimated using the following formula:

$$longest\ diameter\ x\ shortest\ diameter2^2\ /\ 2$$

Patient samples. HCC tissue samples were used to generate tissue microarrays, which were used for immunohistochemistry and ISH studies. All experiments were performed in accordance with relevant guidelines and regulations and all study procedures and protocols were approved by the Institutional Review Boards of Fundeni Clinical Institute. Furthermore, we collected blood samples from patients

treated by regorafenib as standard of care (HCC) (using the DFCI Protocol No.: 13-416 "*Collection of Specimens and Clinical Data for Patients with Known or Suspected Solid Tumors*", PI, Dr. Juric, MGH) or in a phase I clinical trial of regorafenib monotherapy in acute myeloid leukemia (AML) patients (ClinicalTrials.gov Identifier: NCT03042689, PI, Hobbs, MGH). Both protocols have been IRB approved, and written consent was obtained from all patients.

Protein analyses. Western blotting was performed using antibodies against Stat1 (D1K9Y), phosphorylated (p)-Stat1 (Tyr701) (D4A7), Stat3 (79D7), p-Stat3 (Ser727) (Cell Signaling Technology, Danvers, MA), and IFN-γ (EPR1108) (Abcam, Cambridge, MA). Multiplexed protein array analysis was performed for protein extracts from whole tumors, using V-PLEX Proinflammatory Panel 1 Mouse Kit, V-PLEX Cytokine Panel 1 Mouse Kit and V-PLEX Chemokine Panel 1 Human Kit (Meso-Scale Discovery, Rockville, MD). For cell supernatant collection, $1x10^6$ of RIL-175 cells were cultured in DMEM medium with 2% FBS and regorafenib was given in a various dose. Supernatant was collected after 36hr of incubation and multiplexed protein array analysis was done using the same kit above.

Flow cytometry analysis. Tumor tissues were extracted from the mice, weighted and minced with a scalpel. Hank's buffered salt solution (HBSS) with 1.5mg/ml of collagenase and hyaluronidase was added to the minced tissues and incubated for 30 min at 37°C. After incubation, digested tissues were passed through the 70μm cell strainer and washed with PBS with 2% BSA. After preparation, cells were suspended in 2ml of PBS with 2% BSA, then counted using a cell counting plate (Kova slide) (if the final concentration of cell exceeded 10^7 cells/ml, a dilution factor was applied). One hundred microliters of this suspension were used per subsequent flow cytometry staining. The relative count for each lymphoid and myeloid cell was established after specific immunostaining and respective cell subsets counts were established using an LSR-II flow cytometer. The respective cell subsets counts were multiplied accordingly to obtain the count in the initial suspension volume (usually 2ml of PBS

Shigeta K, *et al. J Immunother Cancer* 2020; 8:e001435. doi: 10.1136/jitc-2020-001435

with 2% BSA), then divided by the total weight of the tissue (in gram) to derive the total cell count per gram of tissue (relative quantity).

RNAseq analysis. Three samples were collected from mice treated with regorafenib (10mg/kg) plus anti-PD1 antibody combination for 8 days and from control treated mice. Mice were sacrificed and total RNA was isolated from the freshly isolated tumors tissues using Qiagen kits. Total RNA was sequenced at Molecular Biology Core Facilities, Dana Farber Cancer Institute (Boston, MA). Two samples from each group (which showed response in the combination therapy group and progressive disease in the control group) were used for GSEA and differential expression analyses.

Tissue microarray (TMA). Two tissue cores from different areas of each tumor were punched and transferred to a TMA recipient block. In brief, tissue cylinders with a diameter of 1.2mm each were collected from selected tumor tissue blocks using a homemade semi-automated precision instrument and brought into empty recipient paraffin blocks. Four μm sections of the resulting TMA blocks were transferred to an adhesive coated slide system (Instrumedics Inc., Hackensack, NJ).

Immunohistochemistry. Resected tumor tissues were either snap frozen or embedded in OCT compound. Tissue cryosections were thawed, air-dried, and then rehydrated in PBS followed by incubation with 10% normal donkey serum to reduce nonspecific background staining. Tissues were then incubated with various primary antibodies at 4°C overnight in humidified chambers. After immunostaining with the primary antibody, specimens were incubated with combinations of fluorescent (FITC, Cy3, and Alexa 647) conjugated anti-rat, -hamster, and -rabbit secondary antibodies, as necessary (Jackson ImmunoResearch). Analysis was performed using a laser-scanning confocal microscope (Olympus, FV-1000).

Table S1: Pharmacokinetic parameters measured in plasma and tumor tissue after oral treatment with regorafenib in mice with HCC and liver damage.

Dose (mg/kg)	Regorafenib							
	Plasma				Tumor			
	AUC (ug h/L)	AUC_{norm} (kg h/L)	C_{max} (ug/L)	T_{max} (h)	AUC (ug h/L)	AUC_{norm} (kg h/L)	C_{max} (ug/kg)	T_{max} (h)
5	1450	0.290	108	4	2320	0.465	191	4
10	2140	0.241	201	1	4670	0.467	291	7
10 (+aPD1)	2160	0.216	190	1	5580	0.558	390	4
20	5750	0.287	485	4	7930	0.396	575	4

Dose (mg/kg)	M-4							
	Plasma				Tumor			
	AUC (ug h/L)	AUC_{norm} (kg h/L)	C_{max} (ug/L)	T_{max} (h)	AUC (ug h/L)	AUC_{norm} (kg h/L)	C_{max} (ug/kg)	T_{max} (h)
5	363	0.0749	21.7	7	1040	0.209	52.8	4
10	663	0.0684	40.5	7	2100	0.210	117	7
10 (+aPD1)	587	0.0606	34.2	7	2350	0.235	145	7
20	1480	0.0763	124	7	3780	0.189	214	7

Shigeta K, *et al. J Immunother Cancer* 2020; 8:e001435. doi: 10.1136/jitc-2020-001435

Table S2: Fold change in gene expression between combination therapy and control evaluated by RNA sequencing analysis in whole HCC tissue.

**Gene**	**Regorafenib + anti-PD-1**	
	**Log Fold change**	**FDR**
Cxcl12	2.936301	3.61E-18
Cxcl1	2.465158	3.11E-12
Cxcl13	2.691937	7.64E-06
**Cxcl10**	**0.890427**	**0.002573**
Cxcr6	1.243915	0.003299
**Cxcr3**	**1.333189**	**0.007156**
Cxcl3	0.944457	0.050482
Cxcl11	0.697943	0.185514
Cxcr2	0.522997	0.319418
Cxcl16	0.368327	0.346403
Cxcl17	0.585055	0.421899
Cxcr4	0.321818	0.433014
Cxcl2	0.55163	0.446823
Cxcl14	0.174668	0.670036
Cxcl5	0.091492	0.765404
Cxcl9	0.021916	0.96147

Shigeta K, *et al. J Immunother Cancer* 2020; 8:e001435. doi: 10.1136/jitc-2020-001435

Supplemental Figure Legend

Fig. S1: Functional consequences of vascular normalization after treatment with regorafenib and PD-1 blockade in RIL-175 HCC model. (**A**) Mice with established (4-5mm in diameter) HCCs were treated with anti-PD1 therapy (P) alone or in combination with 5mg/kg (R5), 10mg/kg (R10) or 20mg/kg (R20) regorafenib versus control (C) (n=8 mice per group, 1-week treatment). Quantitative analysis of IF data showed increased microvascular density (MVD) only after R10+P treatment. (**B**) IF analysis for CA-IX showed a corresponding trend for decreased hypoxia after R10+P at this early time-point (p=0.10 from ANOVA test). (**C**) Representative IF confocal microscopy images of tissue hypoxia using CA-IX staining (red) in RIL-175 tumor sections. Scale bars, 50µm. (**D**) Significant increase in CD31+ vessel density in the RIL-175 mouse model of HCC after 12 days of treatment with R10 alone or R10+P. (**E**) Representative IF confocal microscopy images of tumor vessels using CD31 staining (green) and tumor hypoxia using CA-IX staining (red) of RIL-175 tumor sections. Scale bars, 100µm. (**F**) Pharmacokinetic (PK) analysis of regorafenib in plasma (upper panel) and tumor tissue (lower panel). No difference was seen in plasma maximum concentration (Cmax) of regorafenib between R10 alone versus R10+P. In contrast, Cmax of regorafenib in the tumor tissue was higher in combination group (the difference at 4 hr reached statistical significance, $p<0.05$) than R10 group (n=4-5 mice). Represent geometric mean and geometric standard deviation.

Fig. S2: Effects of combining an optimal dose of regorafenib (10mg/kg) with anti-PD-1 therapy on immune environment in RIL-175 murine HCC model. (**A, B**) Regorafenib (10mg/kg daily, R10) combined with anti-PD-1 therapy (P) did not significantly change the number of CD11b+Gr1+ myeloid-derived suppressor cells (MDSCs) (**A**) or M2-type tumor-associated macrophages (**B**) per gram RIL-175 tumor tissue measured by flow cytometry (n=11-13 mice, 12 days of treatment). (**C**) Representative high-power IF confocal microscopy images of CD8 T-cell distribution (in red) in whole

RIL-175 tumor tissue sections; CD31 staining of vessels in green and DAPI counterstaining in blue. Scale bars, 50μm. (**D**) IFN-γ expression in RIL-175 tumors was increased after R10/P combination therapy in whole tissue lysate (Western blot analysis, n=10-13 samples; below, ratio of IFN-γ and β-actin by densitometry analysis). (**E**) Analysis of transcriptional changes in RIL-175 HCCs after combination R10+P versus C using RNA seq analysis: Heat map analysis showed clustering of differentially upregulated and downregulated genes in the combination treatment group compared to control, including increase in CXCL10 and CXCR3 (see **Table S1**). (**G**) Similar trends for gene expression changes with a published poor prognostic angiogenic gene (PPAG) signature (see Tian et al., *Nature* 2017, ref. [20]). (**B**)

Fig. S3. Effects of combining an optimal dose of regorafenib with anti-PD-1 therapy on CXCL10 and CXCR3 in RIL-175 HCC mouse model. (**A**) Regorafenib (10mg/kg daily, R10) combined with anti-PD-1 therapy (P) shows a tendency for increase in CXCL10 protein concentration in whole RIL-175 tumor tissues compared to control (C), evaluated by ELISA (n=4 mice). (**B, C**) R10 alone and R10+P combination therapy decrease STAT3 activity by Western blot analysis (normalized to total STAT3, right) in RIL-175 HCC tissue (n=4 mice). Data represent mean values. (**D**) Correlation between CXCL10 and other immune related genes in The Cancer Genome Atlas (TCGA) analysis. CXCR3, STAT1, CD8A, and IFNG were positively correlated with CXCL10 expression in human HCC samples using Pearson correlation coefficient. (**E**) Treatment with regorafenib increases CXCL10 expression by HCA1 murine HCC cells *in vitro* after 36hr, in a dose-dependent manner (n=6). Culture medium alone was used as negative control (NC). (**F**) *In vitro* treatment of RIL-175 murine HCC cells with STAT3 inhibitor (LL12) at a dose of 2μM for 36hr increased CXCL10 protein concentration measured by ELISA of (n=6). Culture medium alone was used as NC. (**G, H**) R10+P therapy increases the number of CD8+CXCR3+ measured by flow cytometry in tumor tissue (**G**) but not the spleen

tissue (**H**) from HCC-bearing mice versus control (C) (n=5, p=0.045). (**I**, **J**) Regorafenib increases protein CXCL10 expression in a dose dependent manner, as measured by Western blot analysis of while RIL-175 HCC tissue at 6hr after *in vivo* with R10 and R20 (**I**); Quantitation of Western blot data (n=3) is shown in **J**.

Fig. S4: Dose-dependent efficacy of regorafenib when combined with PD-1 blockade in orthotopic grafted and spontaneous HCC models in mice.

(**A, B**) Survival experiment data from RIL-175 orthotopic mouse model. **A**; Lower dose of regorafenib 5mg/kg (R5) and anti-PD-1 blockade combination did not have additional survival benefit compared to regorafenib 10mg/kg (R10) (n=10-13 mice). **B**; Increasing the dose of regorafenib to 20mg/kg (R20) did not provide a significant survival benefit when combined with anti-PD-1 blockade (P), while regorafenib to 10mg/kg (R10) with P significantly increased survival in the RIL-175 orthotopic HCC model (n=9-10 mice). (**C**) Survival experiment data from HCA-1 highly metastatic orthotopic mouse model. R20+P had no survival benefit compared to R10+P (n=11-13 mice). (**D**) Change of body weight from the treatment in RIL-175 orthotopic mouse model. Data represent Kaplan-Meier distributions (**A-C**).

Shigeta K, *et al. J Immunother Cancer* 2020; 8:e001435. doi: 10.1136/jitc-2020-001435
