# Supplementary figures and images for: Regorafenib combined with PD1 blockade increases CD8 T-cell infiltration by inducing CXCL10 expression in hepatocellular carcinoma
Source: J Immunother Cancer. 2020 Nov 24;8(2):e001435. doi: 10.1136/jitc-2020-001435 (PMC7689089; doi:10.1136/jitc-2020-001435)

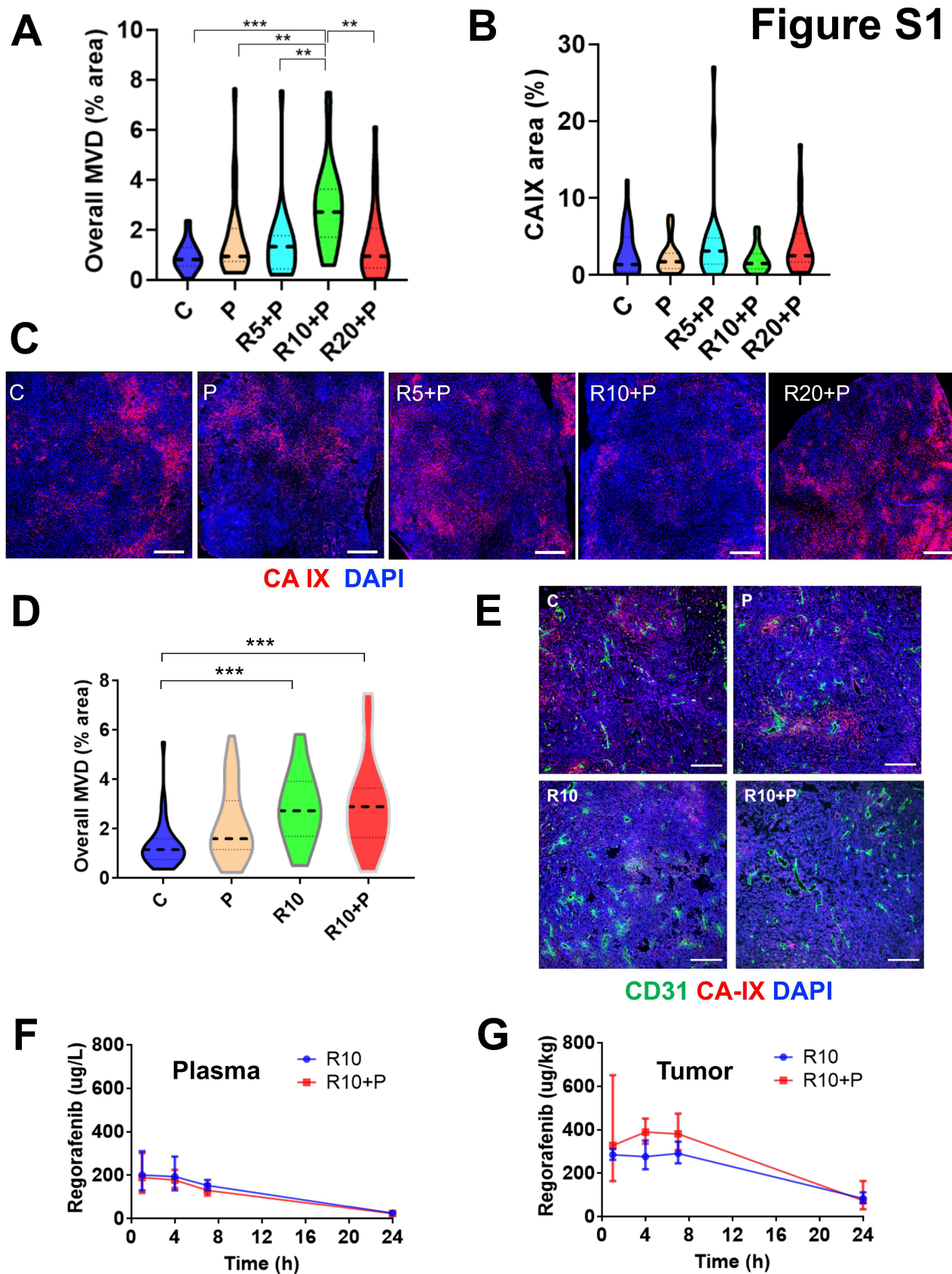

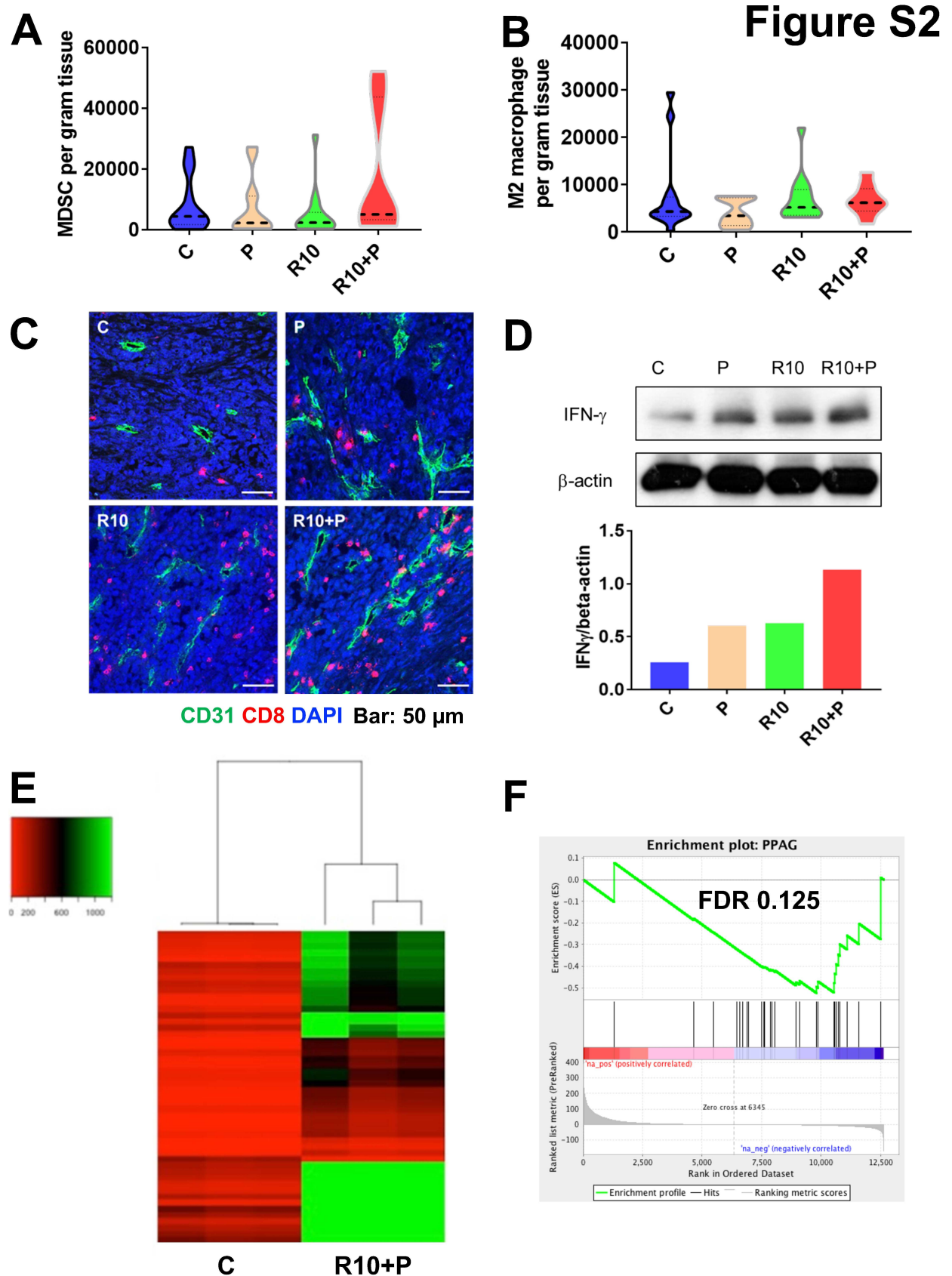

## Figure S3

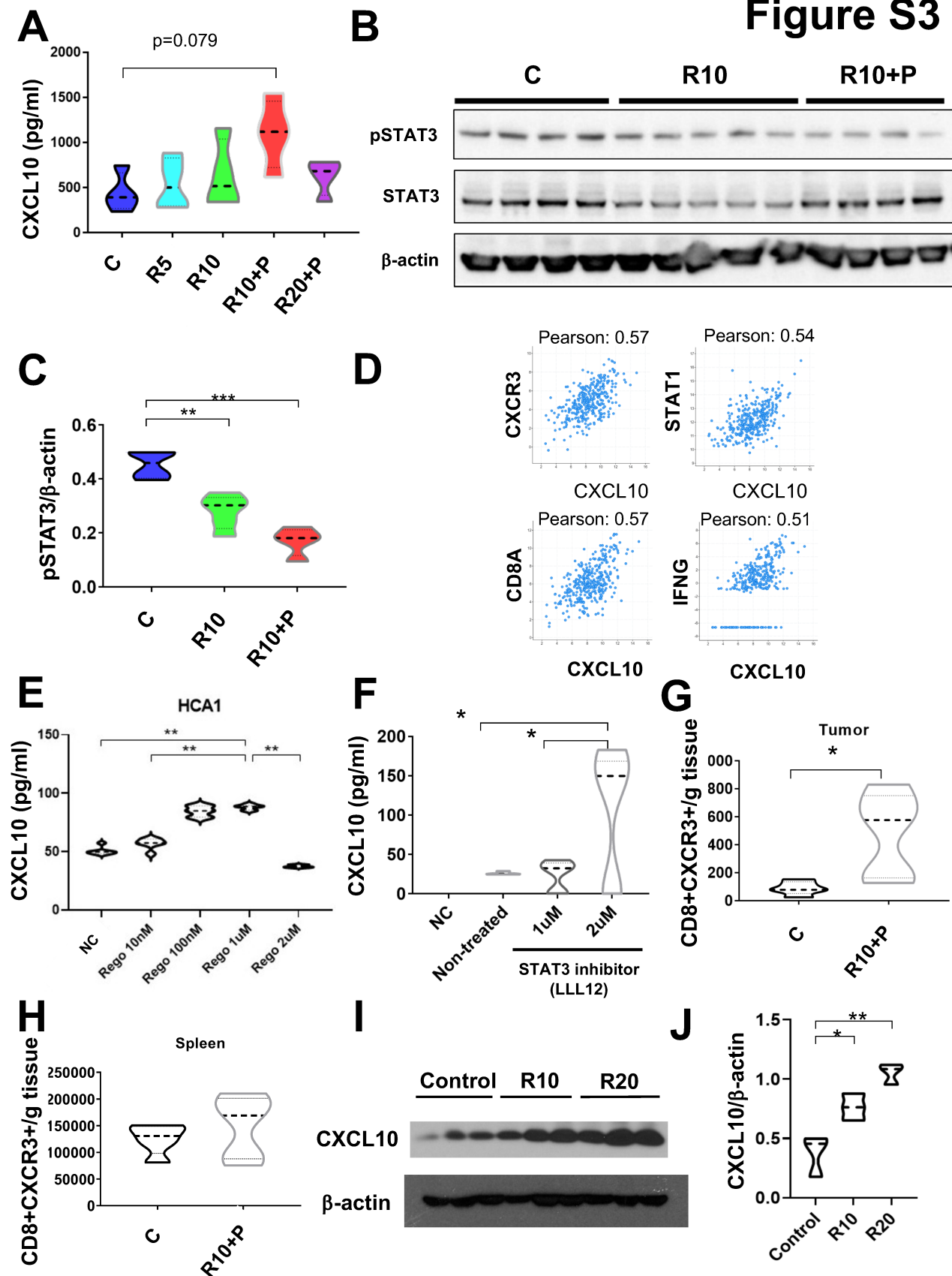

## Figure S4

**A**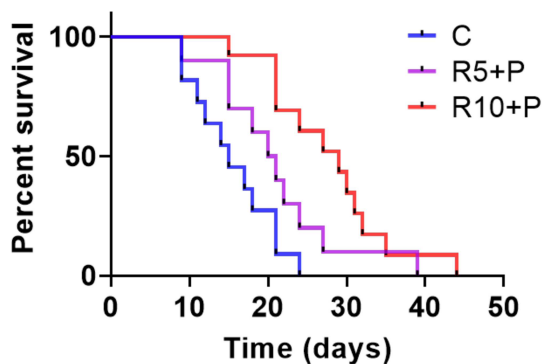**B**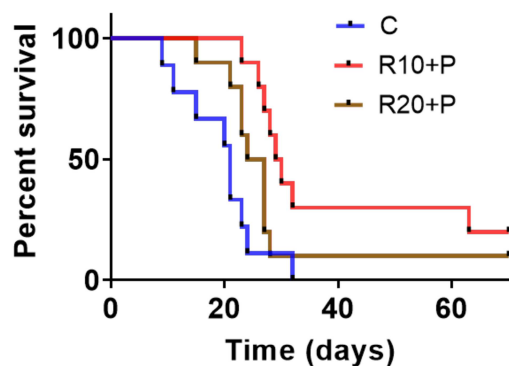**C**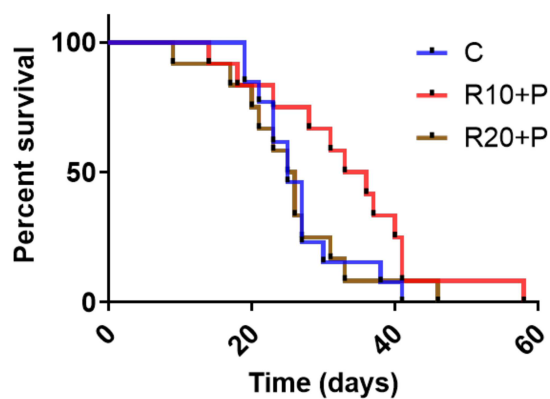**D**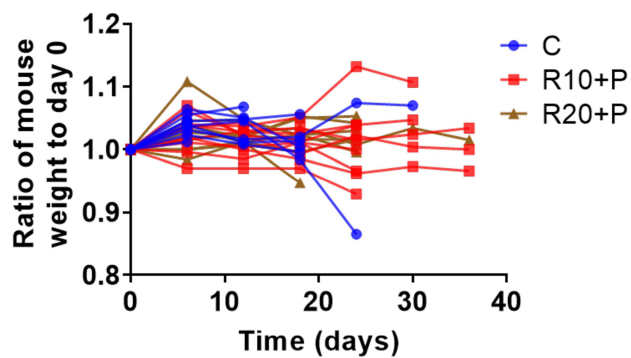

Supplement: Supplementary data [file jitc-2020-001435supp002.pdf]
